# Preventive and Protective Properties of *Zingiber officinale* (Ginger) in Diabetes Mellitus, Diabetic Complications, and Associated Lipid and Other Metabolic Disorders: A Brief Review

**DOI:** 10.1155/2012/516870

**Published:** 2012-11-22

**Authors:** Yiming Li, Van H. Tran, Colin C. Duke, Basil D. Roufogalis

**Affiliations:** Faculty of Pharmacy, The University of Sydney, Sydney, NSW 2006, Australia

## Abstract

*Zingiber officinale* (ginger) has been used as herbal medicine to treat various ailments worldwide since antiquity. Recent evidence revealed the potential of ginger for treatment of diabetes mellitus. Data from *in vitro, in vivo*, and clinical trials has demonstrated the antihyperglycaemic effect of ginger. The mechanisms underlying these actions are associated with insulin release and action, and improved carbohydrate and lipid metabolism. The most active ingredients in ginger are the pungent principles, gingerols, and shogaol. Ginger has shown prominent protective effects on diabetic liver, kidney, eye, and neural system complications. The pharmacokinetics, bioavailability, and the safety issues of ginger are also discussed in this update.

## 1. Introduction

The prevalence of diabetes mellitus has reached epidemic proportions and has affected 6.4% of adults worldwide in 2010 [1, 2]. Diabetes mellitus is characterised by chronic hyperglycemia resulting from impaired insulin action/secretion and is classified into two major categories, type 1 and type 2 [3]. Type 2 diabetes accounts for >90% of diabetes and is associated with metabolic disorder of lipid and carbohydrate [4, 5]. Effective control of hyperglycaemia in diabetic patients is critical for reducing the risk of micro- and macrovascular disease [6–8]. Side effects of the presently available hyperglycaemia agents have impeded their usefulness as antidiabetic agents. This has led to continuous effort to explore effective agents for control of diabetes mellitus.

Ginger (*Zingiber officinale*, Roscoe Zingiberaceae) is one of the most widely consumed spices worldwide. From its origin in Southeast Asia and its spread to Europe, it has a long history of use as herbal medicine to treat a variety of ailments including vomiting, pain, indigestion, and cold-induced syndromes [9, 10]. More recently, it was reported that ginger also possessed anti-cancer, anticlotting, anti-inflammatory, and analgesic activities [11, 12]. However, there is less emphasis on the effects of ginger in management of metabolic diseases and their complications. We review here the known effects of ginger on diabetes mellitus, and provide an insight into the active constituents and their mechanisms of action. The pharmacokinetics of ginger and the issue of safety are also discussed.

## 2. Antihyperglycaemic Effects of *Zingiber officinale*

A number of studies have examined the efficacy of ginger in hyperglycaemia control *in vitro *and* in vivo *on cell and animal models and in clinical trials. Investigation of the active principles and mechanism of action have also been undertaken. 

### 2.1. Results from Animal Studies

It was reported that oral administration of an ethanolic extract of ginger (800 mg/kg) significantly decrease fasting blood glucose level after 1 hour treatment in an STZ-type 1 diabetic rat model. The effect peaked after 4 hours, with ginger producing a 24% to 53% reduction in blood glucose at doses ranging from 100 to 800 mg/kg [13]. Another study showed that a single dose of ginger juice prevented 5-hydroxytryptamine-(5-HT-) induced acute hyperglycemia, suggesting the involvement of 5-HT receptors in the glycaemic control. In the oral glucose tolerance test, ginger treatment caused a significant decrease of the area under the curve of plasma glucose levels and an increase of the area under the curve for insulin in STZ-diabetic rats [14]. Long-term treatment with ginger not only affected blood glucose levels, but also decreased serum triglyceride and total cholesterol, increased insulin, and effectively prevented body weight, liver, and kidney weight loss in type 1 diabetic animals [14–16]. 

Type 2 diabetes mellitus is a chronic metabolic disorder associated with low physical activity and high energy intake [17]. A study on high-fat diet-(HFD-) fed rats reported the protective effect of ginger in the development of various parameters of metabolic syndrome, a condition predisposing to a high risk of type 2 diabetes. After treatment with an ethanolic extract of ginger at doses of 100, 200, and 400 mg/kg for 6 weeks, the marked rises in body weight, serum glucose, insulin, total cholesterol, LDL cholesterol, triglycerides, free fatty acid, and phospholipids induced by high-fat diet significantly reduced [18]. In a nicotinamide and low dose STZ-induced type 2 diabetic rat model, oral administration of ginger powder (200 mg/kg) alleviated signs of metabolic syndrome, including decrease of blood glucose, total lipid, and an increase in total antioxidant level [19]. In a combined high-fat diet and STZ-induced type 2 diabetic animal model, better glucose tolerance and enhanced serum insulin concentration were observed in ginger-treated diabetic rats [20]. Furthermore, the major pungent component in ginger, [6]-gingerol (100 mg/kg bw) significantly decreased fasting blood glucose and improved glucose tolerance in db/db type 2 diabetic mice and lowered plasma triglyceride, total cholesterol, free fatty acid, low-density lipoprotein, and plasma insulin levels [21].

In normal animals, the effects of ginger were not always consistent. It was reported that a glucose-lowering effect was observed 1 hour after administration of ginger extract [13]. However, a standardised dry ginger ethanol extract (containing 1.9 w/w of gingerol) formulated in corn oil EV.EXT 33 (25, 50 and 100 mg/kg) did not show any effect on blood glucose in normal rats at 3 hours postdose [22]. Furthermore, in rats fed with fresh squeezed ginger juice for 6 weeks, neither blood insulin nor cholesterol and triglyceride were affected [14].

### 2.2. Results from * In Vitro* Studies

An *in vitro *study verified that an ginger ethyl acetate extract stimulated glucose uptake and GLUT4 expression in L6 myotube cell surface, reduced lipid content in 3T3 adipocyte, and inhibited protein glycation [23]. 

Although it is generally accepted that gingerols, the major pungent component in ginger, are the most prominent biologically active components, direct evidence for the action and mechanism of gingerols in improving glucose homeostasis have not been reported until recently. It was found that the moderate polar portion of ginger extract containing mainly gingerols, particularly (*S*)-[6]- and (*S*)-[8]-gingerol, promoted glucose uptake significantly in L6 cultured rat skeletal muscle cells. This action of gingerols was attributed to facilitation of insulin-independent glucose uptake by increasing translocation of glucose transporter GLUT4 to the muscle cell plasma membrane surface, together with small increases in total GLUT4 protein expression [24]. It was also reported that [6]-gingerol promoted glucose uptake in insulin responsive 3T3-L1 adipocytes [25].

### 2.3. Results from Clinical Trials

Limited clinical studies have been conducted to investigate the potential beneficial effects of ginger in patients. After consuming 3 g of dry ginger powder in divided dose for 30 days, significant reduction in blood glucose, triglyceride, total cholesterol, LDL, and VLDL cholesterol was observed in diabetic patients [26]. However, in patients with coronary artery disease taking 4 g of ginger powder for 3 months, neither blood glucose nor lipid was altered [27]. Such discrepancy of results may be attributed to the variation in chemical composition of the administered ginger extracts, depending on the preparation method, product origin, or storage duration [28, 29]. Clearly more clinical studies are required to evaluate the effectiveness and the pattern of effects of ginger in human subjects with diabetes and metabolic disorders.

## 3. Mode of Action of *Zingiber officinale* on Glycaemic Control 

### 3.1. Ginger Inhibits Enzymes in Carbohydrate Metabolism

The key enzymes controlling carbohydrate metabolism associated with hyperglycaemia and type 2 diabetes are *α*-amylase and *α*-glucosidase. An *in vitro *enzyme inhibition study was conducted on successive extracts of ginger with hexane, ethyl acetate, methanol, 70% methanol-water, and water. The ethyl acetate extract showed the highest *α*-glucosidase and *α*-amylase inhibitory activity, with IC_50_ values of 180 mg/mL and 980 mg/mL, respectively. No effects were observed in other extracts. The action of ginger against these two enzymes was found to be correlated with the phenolic contents of gingerol and shogaol in these extracts [30]. However, an aqueous extract of Jamaican ginger showed only a slight inhibitory effect on *α*-glucosidase but not *α*-amylase. These results might be related to the low content of the total phenolic compounds in this water extract of ginger [31]. *In vivo* studies on rats showed that after long-term (8 weeks) feeding with ginger, the activities of pancreatic lipase, amylase, trypsin, and chymotrypsin were significantly increased. On the contrary, following a single-dose treatment of ginger, the activities of these enzymes were lowered in pancreas, while in intestinal mucosa the amylase and sucrase was stimulated [32]. 

### 3.2. Ginger Increases Insulin Release and Sensitivity

Diabetes mellitus is characterised by defects in insulin release or/and insulin sensitivity. In the STZ-induced type 1 diabetic rat model, the reduction of blood glucose level was observed to be accompanied by decreased insulin concentration [33–35]. Ginger has been shown to modulate insulin release. *In vitro,* ginger extract augmented insulin release in the INS-1 rat pancreatic *β*-cell. It is of interest that this effect was more prominent in the presence of exogenous serotonin. *In vivo, *a glucose tolerance test further confirmed that this ginger extract also enhanced plasma insulin levels in conjunction with lowered blood glucose. In arsenic-induced type 2 diabetic rats, [6]-gingerol showed a protective effect on pancreatic *β*-cells and restored the plasma insulin level [36]. The mechanism underlying this action of ginger may involve interaction with the 5-HT_3_ receptor [37]. It was found that gingerols and shogaol can act on 5-HT_3_ receptor-ion channel complex by binding to a modulatory site distinct from the serotonin binding site, with the potency order [6]-shogaol ≥ [8]-gingerol > [10]-gingerol > [6]-gingerol [38]. The significance of this mechanism remains to be further evaluated. 

Ginger promotes glucose clearances in insulin responsive peripheral tissues, which is crucial in maintaining blood glucose homeostasis. Evidence from *in vitro* studies has demonstrated that ginger extract and its pungent gingerol principles enhanced glucose uptake in cultured L6 rat skeletal muscle cells and 3T3-L1 adipocytes [24, 25, 39].

### 3.3. Ginger Improves Lipid Profiles

Impaired insulin-stimulated glucose metabolism is a common feature in obese and diabetic subjects. It is well established that insulin resistance in peripheral tissues is tightly associated with elevated circulating lipids and tissue lipid accumulation [40]. The mechanism studies showed that excessive free fatty acid and fatty acid oxidation inhibited glucose transport into peripheral tissues, the first rate-limiting step in glucose metabolism [41–43]. A number of studies have demonstrated that ginger possessed prominent lipid lowering effects, and subsequently increased insulin sensitivity. The antiobese and lipid-lowering effects of ginger tested on various animal models are summarised in Table 1. 

Preliminary clinical trials showed that ginger improved lipid profile in diabetic patients [26]. When ginger was used in combination with other herbs, significant physiological changes, including reduction of body weight, skin thickness, waist/hip circumference, were observed accompanied with reduction of serum triglyceride and cholesterol in diabetic and hyperlipidemic patients [44, 45]. 

## 4. Protective Potential of *Zingiber officinale* for Diabetic Complications 

### 4.1. Protective Effect of Ginger on Liver

In a high-fat diet-fed rat model, an ethanolic extract of ginger (400 mg/kg) effectively reduced triglyceride and cholesterol levels in liver. Molecular studies revealed that liver mRNA and protein expression of low-density lipoprotein (LDL) receptor increased, and 3-hydroxy-3-methylglutaryl coenzyme A (HMG-CoA) reductase protein was downregulated. The mechanistic evidence suggested that the lipid homeostasis effect of ginger was partially due to a decrease in cholesterol biosynthesis and in enhanced hepatic uptake of circulating LDL cholesterol [46]. Moreover, ginger treatment suppressed the gene and protein expression of hepatic inflammation markers, TNF*α*, and IL-6 and decreased NF-*κ*B activity [47].

Shanmugam et al. reported that in normal and STZ-induced diabetic rats fed with a diet containing 1% and 2% of ginger powder for 30 days, the blood glucose level in diabetic rats significantly reduced, while normal rats were not affected. Moreover, in diabetic rats treated with ginger, the activities of superoxide dismutase (SOD), catalase (CAT), glutathione peroxidase (GP*x*) and glutathione reductase (GR), decreased in hepatic and renal tissues, which resulted in an increment of glutathione (GSH) level and a decrease of malondialdehyde (MDA) level. Therefore, the production of reactive oxygen species reduced and resultant oxidative damage to liver and kidney were attenuated. From histological examination, the degeneration of liver central vein, hepatocytes, and sinusoids recovered with the ginger treatment. The pathological alterations in kidney were also improved. The protective effect of ginger was associated with decreased oxidative stress [48]. An *in vitro* study showed that zingerone, a metabolite from ginger, inhibited lipid peroxidation in rat liver microsomes at high concentrations (>150 *μ*M) [49].

### 4.2. Protective Effects of Ginger on Kidney

An ethanolic ginger extract was reported to reduce blood glucose level and to restore the total carbohydrates, pyruvate, glycogen, and total protein in kidney tissue of STZ-induced diabetic rats [50]. The same group further investigated the renal cytosolic and mitochondrial enzyme activities. The decreased activities of glucose 6-phosphate dehydrogenase (G6PD), succinate dehydrogenase (SDH), malate dehydrogenase (MDH), and glutamate dehydrogenase (GDH) in diabetic rats were recovered after ginger treatment (200 mg/kg) for 30 days, and the increased lactate dehydrogenase activity was regulated to normal. Histological examination revealed that ginger treatment appeared to regenerate tubules, restore glomeruli, and reduce fatty infiltration [51]. Al-Qattan and his colleagues reported that in STZ-induced diabetic rats injected intraperitoneally with ginger extract for seven weeks, the serum glucose was significantly lowered, and the urine protein reduced to the same level as the normal group. Histological examination clearly revealed that ginger effectively attenuated the progression of structural nephropathy in diabetic rats. Better corpuscular form, capsular wall integrity, and glomerular layout were observed in rats with ginger treatment. More important, less condensation deposits, mesangial fusion, and protein shedding were seen at the renal corpuscle-capsular space region, which indicated that glomeruli perform better than that of the diabetic rat models [52].

### 4.3. Protective Effect of Ginger on the Central Nervous System

A study of ethanolic ginger extract on STZ-induced diabetic rat showed that ginger possessed neuroprotective effects by accelerating brain antioxidative defence mechanisms and downregulating the malondialdehyde (MDA) level. The marked decreased activities of antioxidant marker enzymes, superoxide dismutase (SOD), catalase (CAT), glutathione peroxidase (GP*x*), and glutathione reductase (GR) in diabetic rats were augmented on oral administration of ginger, hence, resulting in reduction of glutathione and malondialdehyde levels in rat cerebral cortex, cerebellum, hippocampus, and hypothalamus [65]. 

### 4.4. Protective Effect of Ginger on the Eye

In the hyperglycemic condition, glucose can be converted to sorbitol and fructose by aldose reductase. Accumulation of polyols results in rapid development of cataract [66]. Compounds isolated from ginger were screened for their aldose reductase inhibitory activities *in vitro*. 2-(4-Hydroxy-3-methoxyphenyl) ethanol and 2-(4-hydroxy-3-methoxyphenyl) ethanoic acid were identified to be of highest potential, with IC_50_ values of 19.2 ± 1.9 and 18.5 ± 1.1 *μ*M, respectively. The inhibitory efficiency corresponds to the length of alkyl side chain and presence of the methoxy group on the aromatic ring. Furthermore, these compounds suppressed sorbitol accumulation in human erythrocytes and lens galactitol accumulation in the galactose-fed cataract rat model [67].

Formation of advanced glycation end products (AGE) is accelerated in hyperglycaemic condition, which will lead to the development of diabetic complications. An *in vitro* test showed that an aqueous extract of ginger at 0.1 and 1.0 mg/mL reduced CML-KLH and MGO-derived AGE products by 60%−80% and glucose-derived AGE products by 50%−60% [68]. In the STZ-induced diabetic rat model, feeding of ginger significantly inhibited the formation of various AGE products, including carboxymethyl lysine in the lens. Moreover, the progression and onset of cataract were delayed [69].

## 5. Pharmacokinetics and Bioavailability of Ginger and Its Major Components

There is a limited but growing information on the pharmacokinetics and metabolism of ginger and its components. A number of studies revealed that the major components of ginger are excreted as metabolites. After oral administration of a single dose of ginger oleoresin (300 mg/kg) to rats, 8-gingerol, 10-gingerol, and 6-shogaol were detected in the plasma as the free forms, while the major pungent principle 6-gingerol mainly existed as glucuronide with a Cmax of 3.86 *μ*g/mL and as the free form at 0.93 *μ*g/mL about 1.2 hours after dosing [70]. When 6-gingerol (50 mg/kg) was given orally to rats alone, approximately 48% of 6-gingerol was excreted into bile as (*S*)-(6)-gingerol-4′-*O*-*β*-glucuronide in 60 hours, and 16% was excreted into urine as other minor metabolites. *In vitro* incubation of 6-gingerol with rat liver generated 6-gingerol glucuronide and two other metabolites, [6]-gingerdiol and 9-hydroxy-[6]-gingerol [71]. Further studies demonstrated that up to eight metabolites were generated, which indicated a more complex metabolism of [6]-gingerol [72]. Similarly, about 78.5% of [6]-shogoal was excrete into bile as metabolites over 48 hours after dosing [73]. Another study conducted in rats showed that the pungent components in ginger can be absorbed rapidly. The maximum plasma concentration of [6]-gingerol (4.24 *μ*g/mL) was achieved after 10 minutes after oral dosing of 240 mg/kg of a ginger extract (containing 53% of [6]-gingerol) and then declined with time in a biexponential pattern, which could be described as a two-compartment open model. The Cmax of [6]-gingerol was seen in the majority of tissues at 30 minutes; the highest value was 534 *μ*g/g in stomach followed by 294 *μ*g/g in small intestine [74]. 

In clinical experiments, when human volunteers took 100 mg to 2 g of ginger extract, there were no free forms of gingerols and shogoal detected in plasma, but these were found as the glucuronide and sulphate conjugates [75]. However, with a more sensitive technique, free forms of 10-gingerol and 6-shogoal, as well as glucuronide metabolites of 6-, 8-, and 10-gingerol and 6-shogoal were identified 1 hour after oral dosing with 2 g of the same ginger extract [76]. It can thus be concluded that gingerols and shogoals are rapidly absorbed in animals and humans and accumulate in a number of tissues and are extensively metabolised in the body. 

## 6. Safety of *Zingiber officinale*

Recently, the safety issue of ginger used as a supplementary medicine has been raised, especially in the specific group of pregnant women using ginger for ease of nausea and vomiting [77, 78]. Ginger is generally considered safe and is recorded in the US pharmacopeia as a tincture to treat stomach upset. In a randomized double-blinded clinical trial conducted in different countries, women who took 1 g to 1.5 g/day of ginger during pregnancy did not show a higher risk of major malformations or other birth defects compared to the general population [79, 80]. The teratogenic potential of ginger was investigated in pregnant rats. The result showed that oral administration of a standardised ginger ethanolic extract EV.EXT 33 at a dose of 1000 mg/kg was well tolerated. After 21 days of pregnancy, there was no difference in the number of fetuses, fetal body weight, and sex ratio between ginger treatment group and vehicle control. Furthermore, the number of foetuses with parietal and occipital bone defect decreased in EV.EXT 33 treated rats [81]. 

The results of toxicological studies showed a broad safety range for ginger usage. The acute toxicity (LD_50_) of methanolic and aqueous ginger extract was 10.25 and 11.75 g/kg, respectively, by oral administration in mice [82]. When ginger ethanolic extract was administrated intraperitoneally, the LD_50_ was 1551 ± 75 mg/kg in mice [13].

The values of LD_50_ of the major pungent components in ginger, [6]-gingerol and [6]-shogoal, were 250 and 687 mg/kg [83]. When ginger is considered for management of components of chronic metabolic syndrome, such as hyperglycaemia or hyperlipidemia, long-term toxicity indeed needs to be investigated. A 35-day toxicity study reported that oral administration of ginger powder up to 2 g/kg once daily did not cause any mortality or abnormal changes of the general condition or haematological parameters in either male or female rats. All the biochemical parameters presented normally except for serum lactate dehydrogenase in males. The male rats also showed a slight but significant decrease of testes weight [84]. On the other hand in diabetic rats, ginger extract was found to enhance male fertility index and sexual organ weight over a 65 day successive treatment [82]. 

## 7. Summary

**Figure 1:**
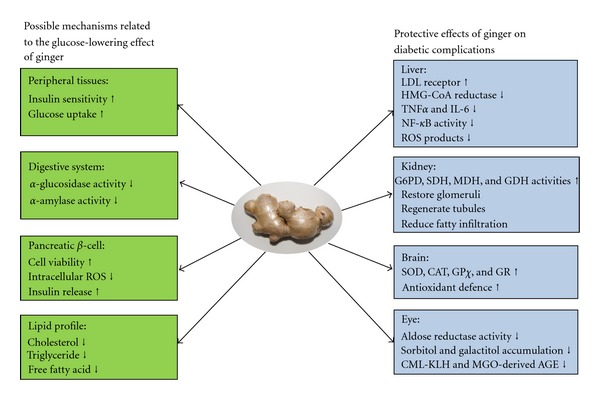
Summary of the mechanism of anti-hyperglycaemic and protective effect of ginger.

*Zingiber officinale* (ginger) shows effective glycaemic control properties in diabetes mellitus. The mechanisms underlying these actions are associated with the inhibition of key enzymes controlling carbohydrate metabolism and increased insulin release/sensitivity, resulting in enhanced glucose uptake in peripheral adipose and skeletal muscle tissues. The prominent lipid lowering effects of ginger also contribute to improving the insulin resistant condition. A protective effect of ginger against diabetic complications is also an important aspect of its benefits (Figure 1). Pharmacokinetic and bioavailability studies provide further information for understanding the metabolism of ginger, especially its pungent principles. Sufficient acute and chronic toxicity studies have demonstrated the broad safety of ginger as a complementary hyperglycaemic control agent.

## Figures and Tables

**Table 1:** Lipid-lowering effect of *Zingiber officinale* in animal models.

Year	References	Ginger preparation	Dosage and administration route	Animal model	Experiment duration	Outcomes	Conclusion
2010	Antihypercholesterolaemic effect of ginger rhizome (*Zingiber officinale*) in rats Elrokh et al. [53]	Aqueous extract	100, 200 and 400 mg/kg p.o.	Hypercholesterolaemic rat	4 week	Serum cholesterol↓ Triglyceride↓ LDL-c↓ HDL-c↑ TC/HDL-c (risk ratio)↓	Significant decrease in all lipid profile parameters, improved risk ratio

2009	Protective effects of ethanolic extract of *Zingiber officinale* rhizome on the development of metabolic syndrome in high-fat diet-fed rats Nammi et al. [18]	Ethanolic extract	100, 200 and 400 mg/kg p.o.	High-fat diet fed rats	6 weeks	Body weight↓ Serum glucose↓ Insulin level↓ Total cholesterol↓ LDL-c↓ Cholesterol↓ Triglycerides↓ Free fatty acid↓ Phospholipids↓	Provides scientific evidence to substantiate the traditional use of ginger in preventing metabolic disorders

2009	Upregulation of mRNA of retinoid-binding protein and fatty acid-binding protein by cholesterol-enriched diet and effect of ginger on lipid metabolism Matsuda et al. [54]	Ginger powder	0.5 g/rat/day in diet	Hypercholesterolemic rats	84 days	Liver retinoid-binding protein mRNA↓ Visceral fat↓	May improve lipid metabolism

2007	Effect of an herbal extract Number Ten (NT) on body weight in rats York et al. [55]	Polyherbal aqueous extract (6-7% ginger)	0.75 and 1.5 g/day p.o.	High-fat diet-fed rat	56 days	Body weight gain↓ Food intake↓ Body fat ratio↓ No difference of serum leptin, metabolites and organ weights (kidney, spleen, liver, and gastrocnemius muscle)	Demonstrated the efficacy in reducing weight gain in rodents

2006	Beneficial effects of* Zingiber officinale *on gold thioglucose induced obesity Goyal and Kadnur [56]	Methanolic and ethanolic ginger extracts	250 mg/kg p.o.	Goldthioglucose-induced obese mice	8 weeks	Body weight↓ Serum glucose↓ Serum insulin↓ Serum lipid↓	Indicates improvement of insulin sensitivity

2005	Effect of ethanolic extract of *Zingiber officinale *on dyslipidaemia in diabetic rats Bhandari et al. [57]	Fresh ginger ethanolic extract	200 mg/kg p.o.	STZ-induced diabetic rat	20 days	Serum total cholesterol ↓ HDL-c↑ LDL- and VLDL-c↓ Serum tryglyceride↓ Phospholipid↓ Blood glucose↓ Liver and pancreas lipid peroxidation↓	Protects tissues from lipid peroxidation, significant lipid lowering activity

2005	Antiobesity actions of *Zingiber officinale *Roscoe Han et al. [58]	Aqueous ginger extract	1 and 3% in diet	High-fat diet-fed mice	8 weeks	*In vitro* pancreatic lipase activity↓ Body weight↓ Parametrial adipose tissue weight↓	Antiobesity effect may be partially due to the inhibition of intestinal absorption of dietary fat

2001	Differential effect of polyherbal, antiobesity preparation OB-200G in male and female mice and monosodium glutamate-treated rats Kaur and Kulkarni [59]	OB-200G, a polyherbal preparation (containing 5% ginger aqueous extract)	0.25, 0.5, 1, and 2 g/kg p.o. twice daily	Male and female Laca mice	21 days	Lower dose (0.25 g/kg): food intake↑ Higher doses: in female mice Food intake in female rats↓	Gender differences involved in mediating antiobesity effect
			0.5 g/kg p.o.	MSG-treated male and female rats	40 days	Body weight↓ Fat pad weights↓ Serum glucose ↑ Ambulatory activity↑	

2001	Investigations on possible serotonergic involvement in effects of OB-200G (polyherbal preparation) on food intake in female mice Kaur and Kulkarni [60]	OB-200G, a polyherbal preparation (containing 5% ginger aqueous extract)	0.5 g/kg (contains ginger aqueous extract 5%) p.o.	Female albino mice of Laca strain with induced hyperphagia	4 hours	Food intake↓	Serotonin is involved in the effect of OB-200G mediated food intake.

2000	Ginger extract consumption reduces plasma cholesterol, inhibits LDL oxidation and attenuates development of atherosclerosis in atherosclerotic, apolipoprotein E-deficient mice Fuhrman et al. [61]	Standardized ginger ethanolic extract (containing 40 mg/g pungent compounds, 90 mg/g polyphenols and 14 *μ*L/g essential oils)	25 and 250 *μ*g/day p.o.	Apolipoprotein E-deficient (E^0^) mice	10 weeks	Plasma cholesterol↓ Triglyceride↓ VLDL & LDL↓ Cellular cholesterol Biosynthesis↓ LDL oxidation and aggregation↓ LDL-associated lipid Peroxides↓	Significant attenuation of the development of atherosclerotic lesions

2000	Antiobesity effect of a polyherbal formulation, OB-200G in female rats fed on cafeteria and atherogenic diets Kaur and Kulkarni [62]	OB-200G, A polyherbal preparation (containing 5% ginger aqueous extract)	400 mg/kg, p.o. twice a day	Female Wistar rats fed with cafeteria and atherogenic diets	40 days	Body weight↓ Body temperature↑ Locomotor activities↑ Serum glucose level↑ Cholesterol↓ (atherogenic diet)	Exhibited antiobesity effect

1998	The protective action of ethanolic ginger (*Zingiber officinale*) extract in cholesterol fed rabbits Bhandari et al. [63]	Ginger ethanolic extract	200 mg/kg p.o.	Hypercholesterolemic rabbit	10 weeks	Serum total cholesterol↓ Serum triglyceride↓ Serum phospholipid↓ HDL-c↑ LDL- and VLDL-c↓ Liver and aorta cholesterol ↓	Indicate ginger is an antihyperlipidemic agent

1993	Cholesterol biosynthesis inhibitory component from* Zingiber officinale Roscoe* Tanabe et al. [64]	Compound (*E*)-8*β*, 17-epoxylabd-12-ene-15,16-dial (ZT) isolated from ginger	25, 50, 100, and 200 mg/kg p.o.	Triton WR-1339-induced hypercholesterolemic mice		Serum cholesterol (50, 100, and 200 mg/kg)↓ Cholesterol biosynthesis in liver↓	Compound ZT has an inhibitory effect on cholesterol biosynthesis
